# Pithyriasis lichénoïde: diagnostic pas toujours aussi facile!

**DOI:** 10.11604/pamj.2018.29.25.10558

**Published:** 2018-01-12

**Authors:** Sara Elloudi, Asmae Lahlou, Mariame Maziane, Salim Gallouj, Fatima Zohra Mernissi, Mouna Rimani

**Affiliations:** 1 Service de Dermatologie-Vénérologie, Centre Hospitalier Universitaire Hassan Hassan II, Route Sidi Hrazem, Fès, Maroc; 2 Centre d’Anatomopathologie Hassane, Rabat, Maroc

**Keywords:** Pityriasis lichenoides, indelible scars, cyclins, phototherapy, Pityriasis lichénoïde, cicatrices indélébiles, cyclines, photothérapie

## Abstract

Le pityriasis lichénoide est une dermatose inflammatoire rare du sujet jeune évoluant par poussées spontanément régressives sur le tronc et les membres. Nous rapportons un cas particulier de pityriasis lichenoide, dont le diagnostic était redressé par l'aspect cicatriciel des lésions et dont l'évolution était favorable sous cycline et photothérapie.

## Abstract (en)

Pityriasis lichenoides is a rare inflammatory dermatosis occurring in young subjects. It evolves into rashes on the trunk and the limbs which regress spontaneously. We here report a particular case of pityriasis lichenoides whose diagnosis was adjusted due to the scar appearance of the lesions. The patient was treated with cyclin and phototherapy, with a favorable outcome.

## Introduction

Le pityriasis lichénoide (PL) est une dermatose inflammatoire rare qui atteint préférentiellement les enfants et les adultes jeunes, évoluant par poussées spontanément régressives sur le tronc et les membres. On distingue la forme aigue varioliforme et nécrotique, d'une forme chronique papulosquameuse. La forme leucomélanodermique peut survenir d'emblée ou succéder aux deux formes précédentes. Ces types de lésions peuvent également s'associer, ce qui conduit à envisager des formes de passage entre ces trois variantes d'une même pathologie. Il n'existe pas de traitement consensuel pour cette affection. Nous rapportons un cas clinique particulier de pityriasis lichenoide, dont le diagnostic a été redressé par l'aspect cicatriciel des lésions, et dont l'évolution était favorable sous cycline et photothérapie.

## Patient et observation

**Figure 1:**
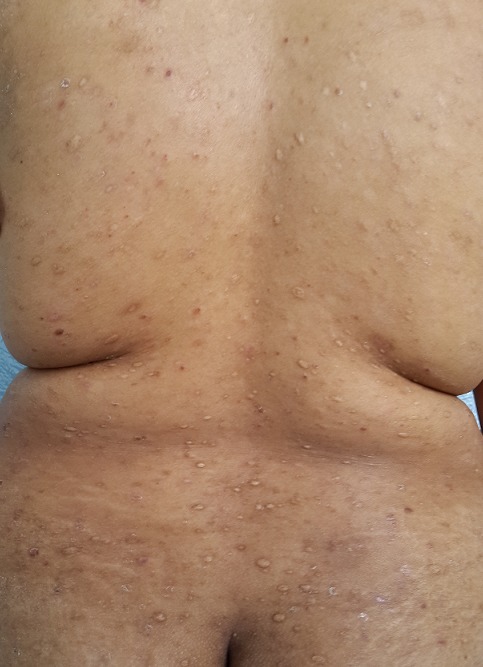
Macules et papules hypochromiques et pigmentées, anétodermique par endroit au niveau du dos

**Figure 2:**
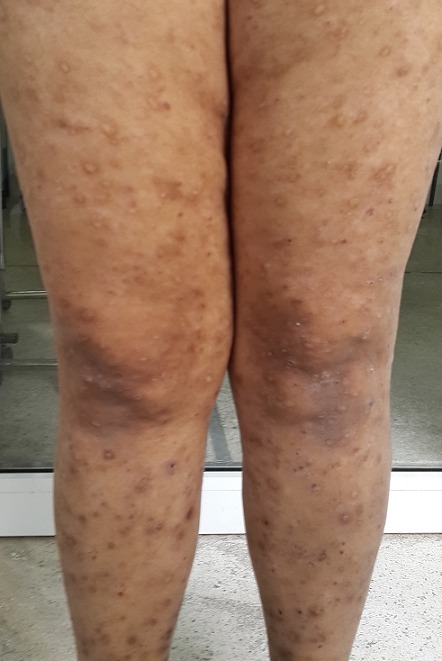
Des petites érosions et macules pigmentées au niveau des membres inférieurs

**Figure 3:**
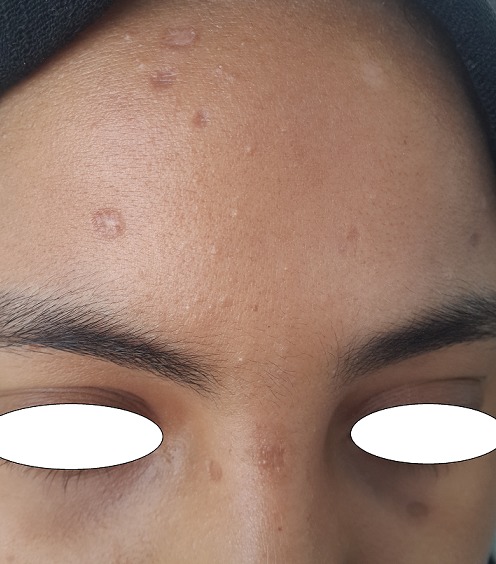
Cicatrices varioliformes au niveau du visage

**Figure 4:**
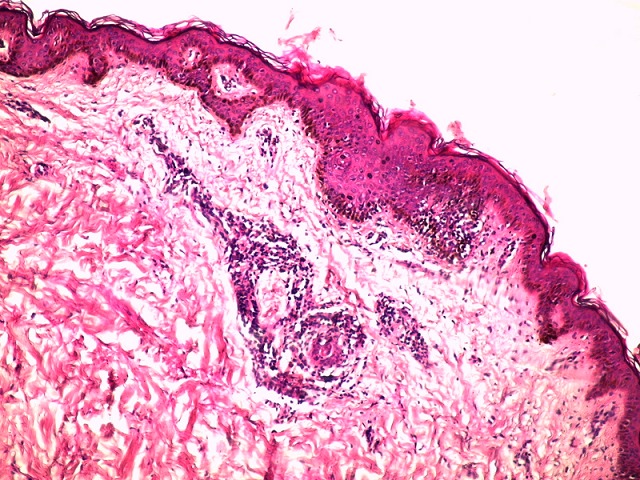
Coloration HES G x 100 réaction lichénoïde + manchons inflammatoires périvasculaires du derme superficiel

**Figure 5:**
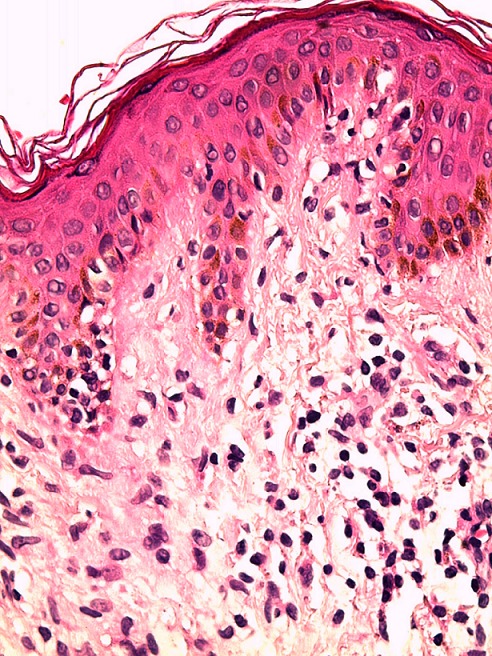
Coloration HES G x 400 quelques cellules basales vacuolisées et exocytose basale à petits lymphocytes

**Figure 6:**
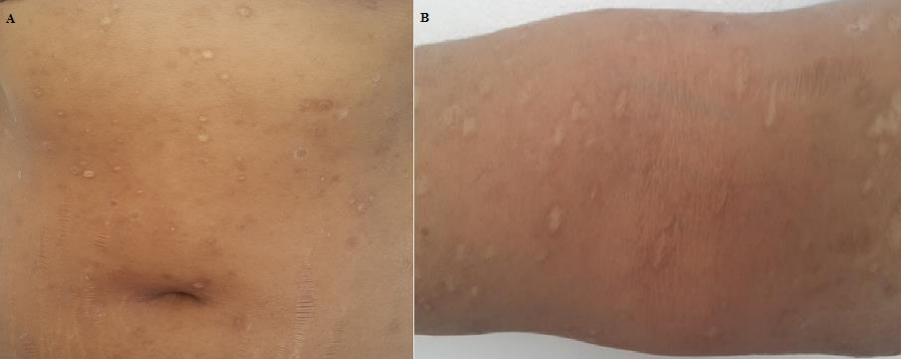
(A, B) régression des poussées avec absence de lésions actives après traitement et amélioration des lésions cicatricielles

Une jeune fille de 17 ans, suivie pendant cinq ans pour une éruption non prurigineuse du corps prise en charge pendant 2 ans comme une dermatose bulleuse type dermatite herpetiforme, mise sous disulone sans amélioration. La patiente consultait notre service pour la persistance des lésions laissant place à des cicatrices pigmentées et hypochromiques inesthétiques disséminées à tout le corps. L'examen trouvait une patiente apyrétique en bon état général, l'examen dermatologique trouvait de multiples macules et papules hypochromiques et pigmentées, anetodermiques (Figure 1, Figure 2), des petites érosions et papules surmontées de vésicules et de squames par endroit disséminées à tout le corps, quelques cicatrices varioliformes au niveau du visage et corps (Figure 3), Les muqueuses étaient saines. Les aires ganglionnaires étaient libres et il n'y avait pas d'hépatosplénomégalie. Le bilan biologique était normale except une anémie inflammatoire. La biopsie cutané concluait à un PL chronique au stade cicatriciel avec anétodérmie secondaire en montrant un revêtement épidermique plutôt subatrophique et d'aspect ondulé surmonté d'une couche cornée épaissie et compacte. Les couches basales du corps muqueux de Malpighi présente une pigmentation mélanique de répartition inégale et par places exagérée. Le derme papillaire plutôt fibroedémateux abrite très focalement de discrets manchons lymphocytaires à disposition périvasculaire avec de très rares hématies extravasées (Figure 4). Sur les multiples niveaux de coupe réalisés, on observe très focalement une discrète réaction lichénoïde ponctuelle avec vacuolisation des cellules basales associée à une discrète exocytose à petits lymphocytes (Figure 5). Il n'est pas observé de kératinocyte apoptotique. La coloration à l'Orcéine montre un réseau élastique légèrement réduit au niveau du derme papillaire et du derme réticulaire superficiel. La patiente était mise initialement sous corticothérapie orale à raison de 0,5mg/kg/jr pendant 1 mois sans nette régression des lésions actives, puis mise sous cycline pendant 6 mois avec raréfaction des poussées (Figure 6 A,B). Les lésions cicatricielles ont bien répondu à la photothérapie UVB généralisé à spectre étroit (TL01) avec un recul de 18 mois (Figure 6 A,B))

## Discussion

Le PL est une dermatose inflammatoire d'étiologie inconnue, caractérisé par une éruption constituée de nombreux éléments maculopapuleux et squameux. Trois formes cliniques distinctives sont décrites : la forme chronique décrite en 1889 par juliusberg [1] dont la lésion élémentaire est une papule érythémateuse surmontée de squame se détachant en bloc à la curette réalisant ainsi le classique signe de la squame en pain à cacheter. L'évolution peut être prolongée entrecoupées de phase de rémission. La forme aigue varioliforme, individualisée en 1916 par Mucha puis en 1925 par Habermann [2] comporte des éléments nécrotiques évoluant vers des cicatrices varioliformes. Enfin, la présence de macules achromiantes arrondies non squameuses caractérise la forme leucomélanodermique qui peut survenir d'emblée ou succéder aux deux formes précédentes. Dans notre cas, La présence de cicatrices varioliformes suppose que la patiente avait passée par une forme aigue nécrotique laissant place à ce type de cicatrices, la présence de papules érythémateuses ainsi que la leucomélanodermie suggère la forme chronique au stade cicatriciel. La papulose lymphomatoide est le principal diagnostic differentiel de la forme aigue du PL, alors que la forme chronique peut faire discuter le psoriasis en goutte, une toxidermie lichénoide, une syphilis secondaire, une morphée en goutte ou bien un lichen scléroatrophique. Dans notre cas, Ces diagnostics ont été éliminés avant de conclure à un PL. L'aspect histologique dépend du stade évolutif de la maladie. Dans la forme aigue varioliforme, la nécrose kératinocytaire est au premier plan, et l'atteinte vasculaire est plus marquée avec des images de vasculite leucocytoclasique. Le derme superficiel est très oedémateux. Dans la forme chronique, le diagnostic peut être difficile car les lésions histologiques peuvent être discrètes et peu spécifiques, elles justifient une confrontation anatomo-clinique attentive pour parvenir au diagnostic comme c'était le cas chez notre patiente. L'épiderme est discrètement acanthosique, surmonté de zones de parakératose compacte, sèche avec quelques foyers d'exocytose lymphocytaire, une spongiose inconstante et une vacuolisation basale. Le derme superficiel est le siège d'infiltrats de cellules mononucléées à tropisme périvasculaire [3].

L'étiopathogénie de cette affection n'est pas clairement élucidée, plusieurs auteurs ont évoqué une hypersensibilité à un agent bactérien, parasitaire ou viral sur certains cas d'association à une infection évolutive ou de guérison après traitement d'un foyer infectieux sous-jacent [4]. L'origine toximédicamenteuse était également rapportée dans de rares cas [5]. Plusieurs traitements ont été essayés au cours du PL tel que les macrolides, les cyclines la pénicilline ou la rifampicine en raison de différentes hypothèses étiopathogéniques proposées. Des résultats satisfaisants ont été rapporté avec la dapsone [6]. Ce qui pourait expliquer l'atténuation de l'inflammation chez notre patiente sous dapsone avant son hospitalisation. Le methotrexate a également été utilisé avec une certaine efficacité dans des cas de PL aigu et chronique [7, 8]. Les poussées chez notre patientes se sont atténuées sous cyclines. La photothérapie reste le traitement de référence. Nous avons opté pour l'UVB thérapie TL01 comme traitement d'entretien qui a donné de bon résultats. Aucune rechute n'a été noté depuis 18 mois avec amélioration même des cicatrices hypochromiques. Le PL nécessite une surveillance clinique à long terme du fait qu'il existe des cas exceptionnels d'association de PL à un mycosis fongoïde, en particulier chez l'enfant [9].

## Conclusion

Le pityriasis lichénoïde est une dermatose affichante rare dont le diagnostic nécessite une bonne analyse sémiologique des lésions cutanées. Une prise en charge précoce peut prévenir les lésions cicatricielles indélébiles qui peuvent engendrer un fort retentissement social, psychologique et physique. Nous pensons que le traitement par les cyclines puis la photothérapie UVB généralisée peuvent stopper l'évolutivité de la maladie et améliorer les cicatrices hypochromiques disséminées.

## Conflits d’intérêts

Les auteurs ne déclarent aucun conflit d'intérêts.
